# The longitudinal study for the work-related factors to job performance among nurses in emergency department

**DOI:** 10.1097/MD.0000000000014950

**Published:** 2019-03-22

**Authors:** Fu-Li Chen, Kuan-Chen Chen, Shy-Yang Chiou, Peter Y. Chen, Man-Li Du, Tao-Hsin Tung

**Affiliations:** a Department of Public Health, College of Medicine, Fu-Jen Catholic University; b Department of Medical Research and Education, Cheng-Hsin General Hospital, Taipei; c Department of Food Science, National Ilan University, Ilan, Taiwan; d Department of Psychology, Auburn University, AL, USA; e Nursing Department, Huadu District of Guangzhou Maternal and Child Health Hospital (Huzhong Hospital), Guangzhou, China.

**Keywords:** contextual performance, emergency department, follow-up study, job performance, nurses, task performance

## Abstract

To explore the relationship between baseline information, personal factors, working characteristics and job performance among nurses in emergency department in northern Taiwan.

Two-hundred twenty-two nursing staff were interviewed repeated with structured questionnaires for data collection in 3 time points (From August to September, 2008, from February to March, 2009, and from November to December, 2009). The generalized estimating equation (GEE) is used to test the relationship between the domains of independent variables (baseline information, personal factors, working characteristics) and dependent variables (task performance, contextual performance).

The mean age of participants is 30.1 ± 5.1 years. 50.0% are junior college or bachelor degrees. From the GEE model, biological protection (β = 0.17, *P* value = .002) and safety climate (β = 0.24, *P* value < .001) are significantly related to task performance. Contextual performance is strongly affected by safety climate (β = 0.15, *P* value < .001).

To improve the job performance among nurses in emergency department, it should consider personal psychological and environmental factors.

## 1 Introduction

In the current globalized environment, health problems are unimpeded by national borders. The ever-changing nature of healthcare technology and related policies has continually challenged the service efficiencies and quality of healthcare systems and professionals worldwide.^[1]^ Continuing crises in the current healthcare industry have severely degraded the quality and performance of nursing care. Nurses are tasked with various clinical duties such as performing nursing assessments and activities, assisting and executing various medical activities according to doctor instructions, assisting doctors in maintaining patient safety, preventing and controlling infections, providing daily living care to patients incapable of self-care, helping patients adjust to their diseases, and consulting patients and families on healthcare. Providing superior- quality care services is a vital part of the job performance of nurses, which indicates the efficiency of nurses in accomplishing their tasks and responsibilities in patient care.^[2]^

Human resources are the most critical resources for medical facilities. When the employees of a facility achieve their optimal personal performance, they facilitate the facility to attain its overall performance goal as well as ensure that the stakeholders are satisfied with its operation, thereby enhancing the competitive advantages of the facility. In Taiwan, most nurses are hired by medical institutions. In response to changing population structures and prevalent diseases, nurses have become a crucial part of an entire medical team. Stable, exceptional, and high-performing nurses are fundamental for medical teams to provide healthcare services. Insufficiencies in the allocation of clinical nurses have been a persistent problem. Numerous studies have investigated the factors associated with the turnover rate of nurses, and various solutions have been applied to reduce these turnover rates in medical facilities.^[3,4]^ In accordance with the rapid aging of the Taiwanese society, the requirements for clinical nursing care have increased. In addition to continuing to reduce the turnover rate of nurses, the managers of medical facilities must examine and grasp the factors associated with the job performance of nurses and create healthy, perfect, and high-professional-value support environments for nurses by using innovative ideas. Thus, high-performing nurses can be motivated to remain in their jobs, medium-performing and low-performing nurses can be provided with opportunities to improve their performance, and patients can continue to receive high-quality nursing care.^[3]^

Job performance could be divided into task and contextual performance based on the classification system.^[4]^ Task performance was defined as “the effectiveness with which job incumbents perform activities that contribute to the organization's technical core”.^[5]^ Any behavior related to the substantive tasks required by the job was included in this classification.^[6]^ Contextual performance was defined as performance that is not formally required as part of the job but that helps shape the social and psychological context of the organization.^[4]^ Related constructs such as organizational citizenship behaviors and extra-role performance were also included.^[6]^ Several evidence-based studies reported that for among the nurse staff, job performance was positively related to organizational commitment, job satisfaction and personal and professional variables. Job satisfaction and organizational commitment both are essential factors of nurses’ performance. Job performance is also positively associated with personal factors such as years of experience, nationality, gender, and marital status but not level of education.^[7–9]^ To the best of our knowledge, however, few studies have incorporated longitudinal analyses on the factors associated with the job performance of nurses to clarify their causal relationship. Therefore, this study applied a follow-up study design to explore the factors associated with the job performance of the nurses in emergency departments (hereafter referred to as emergency nurses). The results and findings were summarized, and suggestions were provided to medical facility managers for establishing a more favorable support environment, formulating strategies to improve the job performance of nurses, and thereby promoting the quality and performance of the services provided by medical facilities.

## 2 Methods

### 2.1 Research participants

Because private and public hospitals differ in their properties, and local community hospitals and metropolitan hospitals differ in their sizes, a sampling method was applied. A repeated questionnaire was administered to 837 nurses in 4 metropolitan teaching hospitals in northern Taiwan in August to September 2008, February to March 2009, and November to December 2009. After the approval by the institutional review board of each of the hospitals, information regarding the interviewees and contents of the questionnaire surveys was explained to the managers of the hospitals. Our research that involved human subjects was approved by the Institutional Review Board of Taipei City Hospital (Approval TCHIRB-971204-E).

The distribution method of the questionnaires was discussed with the primary supervisors of the hospitals, and the participants were booked and coded. Subsequently, the research assistants packaged the questionnaires and reply envelopes in large envelopes and personally submitted them to the hospitals for the medical personnel in charge to check, accept, and distribute. Each of the participating nurses was awarded with a small gift in acknowledgement of their participation. The completed questionnaires were placed in the envelopes, submitted to the head nurses in the hospitals, and uniformly returned to the researchers by the supervisors of the hospitals.

### 2.2 Measures

This study employed closed-ended questionnaires, which were formulated according to the qualitative interview results and the related studies and research designs. The Cronbach α and confirmatory factor analysis results are listed in Table 1. The contents of the questionnaire are described as follows:

1. Demographic information, which included sociodemographic characteristics (sexes, ages, education levels, and marital statuses), job statuses (units, positions, seniority levels, shifts, duty statuses, working hours, and workloads), and health statuses (medical histories, physical symptoms, and perceived health statuses).
2. Self-protective behaviors, which were divided into injury prevention and the use of assistive or protective gear. A 5-point Likert scale was applied (0 = *never*; 4 = *always*). In addition, the interviewees were asked the reasons why they did not use protective gear, if applicable.
3. Job characteristics, questionnaire items were designed according to the demand– control–support model,^[10]^ which was a revision of the job strain model.^[11]^ According to the job strain model, job control is further divided into 2 constructs, namely skill discretion and decision authority. Skill discretion indicates the skill requirements of employees (e.g., advanced skills, learning new things, and demands for creativity) and the repetitiveness of their job contents. Decision authority refers to the autonomy and discretion exercised by employees in their jobs. A 5-point Likert scale was applied to assess the extent of a participant's agreement with the description provided by each questionnaire item.
4. Safety climate, which was investigated in the health care setting based on the safety climate scales created by Zohar and Luria.^[12]^ A 5-point Likert scale was applied to assess the extent of a participant's agreement with the description provided by each item.
5. Job performance, which was divided into 2 categories in accordance with the findings of previous studies, namely task and contextual performance. The questionnaire items for investigating task performance were based on the core tasks of medical personnel detailed in the US O-net website (http://online.onetcenter.org/). A 5-point Likert scale was applied to assess the extent of a participant's agreement with the description provided by each item.

**Table 1:**
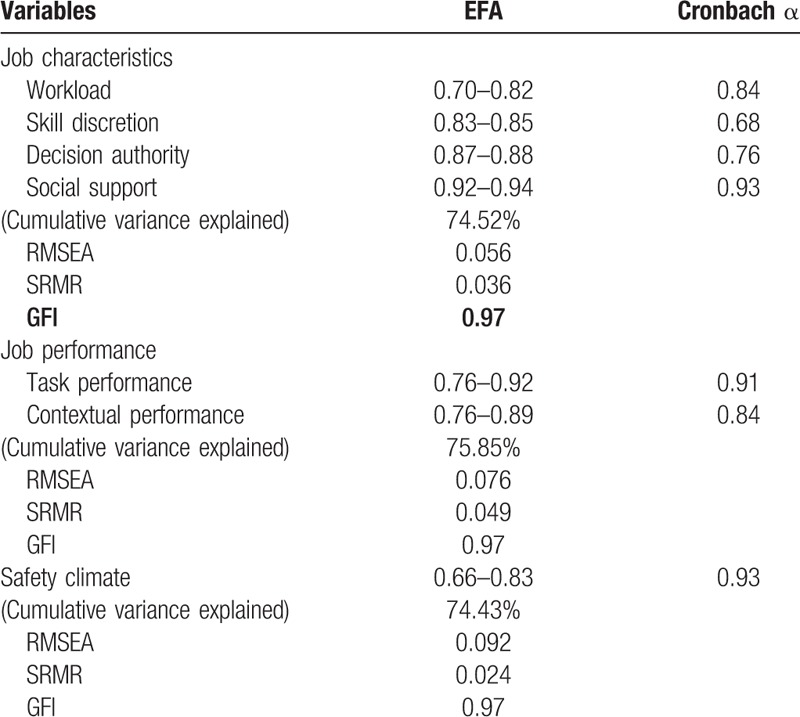
The Cronbach α and confirmatory factor analysis results of the measures.

### 2.3 Statistical analysis

SAS 9.2 was applied to analyze the data with a statistical method appropriate to the goal and structure of the research. A descriptive statistical analysis was conducted to clarify the basic data characteristics and distribution of the valid samples (e.g., mean ± standard deviation, percentage). A repeated measures analysis of variance was performed to clarify the bivariate analysis results of the healthy lifestyles of the emergency nurses. The Pearson correlation was used to analyze the correlations between the continuous variables. Because the measurement variables were time-dependent samples, use of the conventional analysis method would cause a conflict with the hypothesis regarding the independence of the data. Therefore, the causal relationship between the risk factors and death or rejection was explored using the generalized estimating equation (GEE) proposed by Liang and Zeger to meet the requirement of data independence.^[13]^ In the GEE, a set of correlated observations are grouped as a cluster. A working correlation matrix was developed to represent the correlations between the observations in the GEE cluster. The correlation matrix was then incorporated in the GEE, and then a numerical analysis was used to determine the linear model coefficient *β*. The GEE involves aggregating dependent data as clusters, structuring an entire set of data as groups of independent clusters, and processing the clustered data by employing a method similar to the conventional linear model. Therefore, the GEE was used to control the interference factors to clarify the effect of the independent variables (changes in the demographic data, personal factors, and job characteristics) on the dependent variables (task and contextual performance).

## 3 Results

Table 2 lists the basic demographic variables of the interviewees, 99.5% of whom were female. The average age of the interviewees was 30.1 ± 5.13 years. Regarding education levels, 46.8% of the interviewees graduated from junior colleges, and 50.0% graduated from universities or 4-year or 2-year technical programs. More than half of the interviewees were single. In addition, 11.7% of the interviewees were diagnosed with varicose veins, and 9.9% were diagnosed with cardiovascular diseases.

**Table 2:**
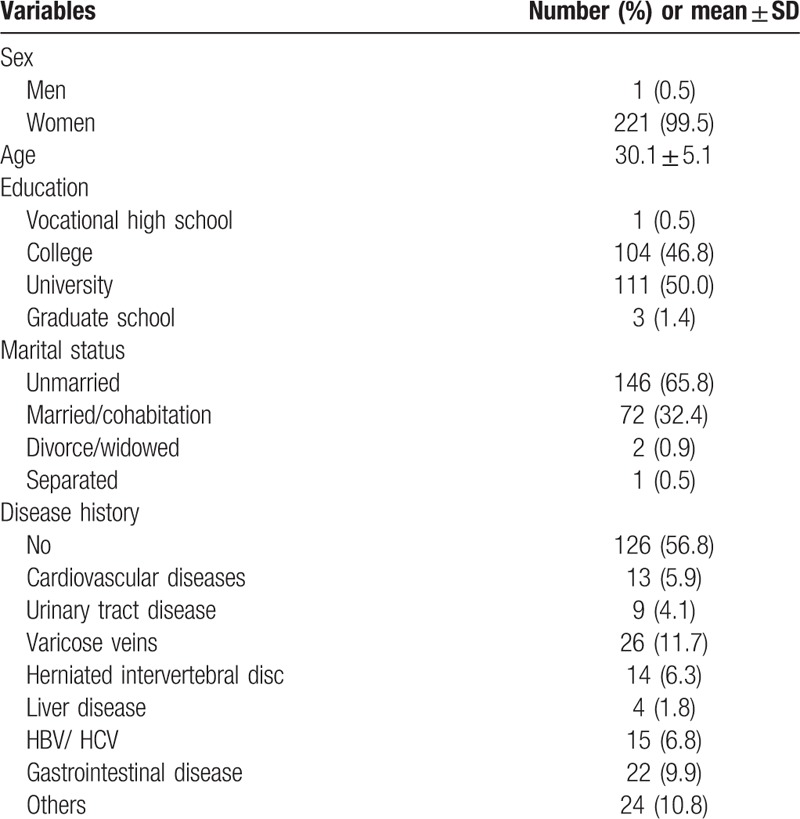
Baseline characteristics among nurses in the emergency department (n = 222).

Table 3 depicts the 3-time questionnaire survey results for the working time, seniority, personal factors, job characteristics, and job performance of the nurses. Examining the results of the repeated measures revealed that regarding the working time and seniority of the nurses, the unit seniority differed significantly between the 3 surveys (*P* = .048). Concerning job characteristics, job demand (*P* = .02), job control (*P* = .04), and safety climate (*P* < .001) differed significantly between the 3 surveys. For job performance, task performance improved significantly over the 3 survey periods, as shown in Table 3 (*P* < .04), but no significant statistical difference between the 3 surveys was observed for contextual performance (*P* = .46).

**Table 3:**
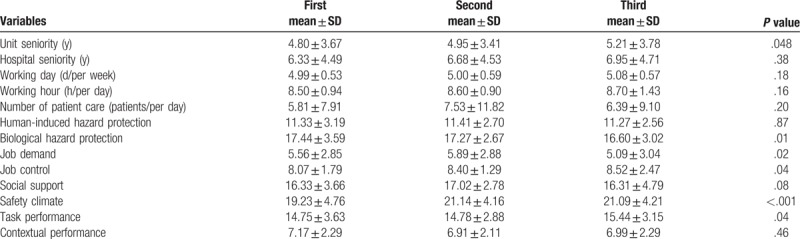
The results of questionnaires among nurses in the emergency (n = 222).

As shown in Table 4, the results of the Pearson correlation analysis revealed that age (*r* = 0 .156), unit seniority (*r* = 0.145), hospital seniority (*r* = 0.172), human-induced hazard protection (*r* = 0 .308), biological hazard protection (*r* = 0.361), job control (*r* = 0.170), and safety climate (*r* = 0.421) were positively correlated to task performance, whereas job demand (*r*Y=Y-0.271) was negatively correlated to task performance. Furthermore, age (*r* = 0.100), diet (*r* = 0.118), human-induced hazard protection (*r* = 0.137), biological hazard protection (*r* = 0.128), job demand (*r* = 0.108), job control (*r* = 0.177), social support (*r* = 0.188), and safety climate (*r* = 0.314) were positively correlated to contextual performance.

**Table 4:**
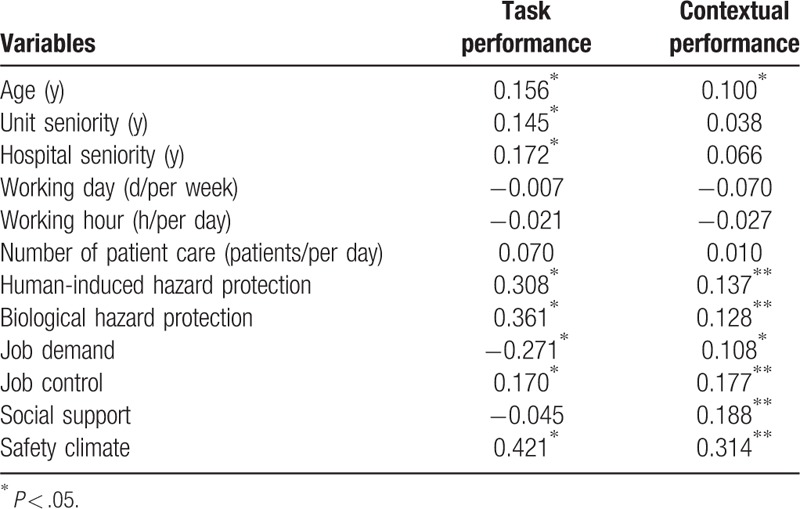
The Pearson correlation of factors related to task performance and contextual performance among 3 investigations (n = 222).

Table 5 lists the GEE analysis results of the factors affecting task performance. After the related risk factors were adjusted, Model I, which concerned the personal physical and psychological factors, revealed that the human-induced (*β* = 0.17, *P* = .01) and biological (*β* = 0.20, *P* = .001) hazard protection of the emergency nurses were significantly associated with their task performance. Model II, which involved the environmental factors and personal factors, revealed that biological hazard protection (*β* = 0.17, *P* = .002) and safety climate (*β* = 0.24, *P* < .001) were significantly associated with the task performance of the nurses.

**Table 5:**
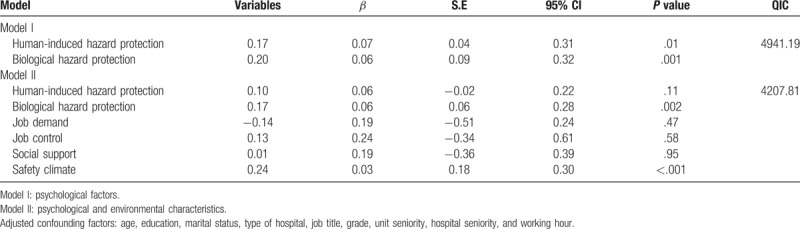
Generalized estimating equation of psychological and environmental characteristics for task performance among nurses in the emergency (n = 222).

Table 6 shows the GEE analysis results of the factors affecting the contextual performance of the nurses. After the related risk factors were adjusted, Model II, which incorporated the environmental factors and personal factors, revealed that safety climate (*β* = 0.15, *P* < .001) was significantly associated with the contextual performance of the nurses.

**Table 6:**
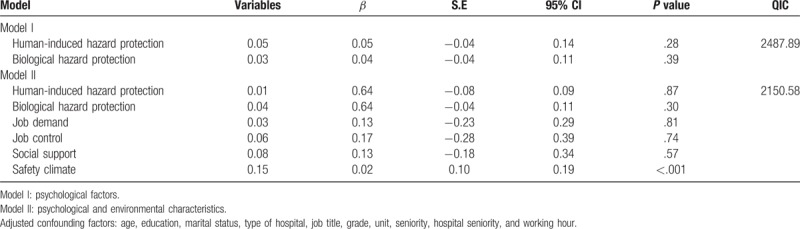
Generalized estimating equation of psychological and environmental characteristics for contextual performance among nurses in the emergency (n = 222).

## 4 Discussion

### 4.1 Clinical implementations

Nurses constitute the majority of professional medical service providers and serve as first-line caregivers to patients. Understanding the factors that affect the job performance of nurses is imperative, and improving the job performance of nurses directly promotes the organizational performance of medical facilities. Task performance indicates the relationship between the work outcome of an individual and the tasks expected of or assigned to the individual by an organization, through which the individual contributes to the core performance of the organization. The proficiency of the individual in job activities can be assessed according to the services or materials required for the individual to directly execute or indirectly support technical procedures.^[14]^ Analyzing the GEE model in the present study revealed that in consideration of the personal physical and psychological factors, the human-induced (*β* = 0.17, *P* = .01) and biological (*β* = 0.20, *P* = .001) hazard protection of the emergency nurses were significantly associated with their task performance. Previous studies have indicated that the factors that affect the self-protective behaviors of nurses against biological hazards are perceived interests, obstacles, and social support, and the psychological constructs of the self-protective behaviors are dependent on the types of health problems being avoided. Therefore, sufficient social support improves the self-protective behaviors of nurses.^[15]^

When both the personal and environmental factors were considered, biological hazard protection (*β* = 0.17, *P* = .002) and safety climate (*β* = 0.24, *P* < .001) were both determined to be significantly associated with the task performance of the emergency nurses. Emergency medical personnel are frequently confronted by the fragile life conditions of critically ill or injured patients who require highly complex and precarious medical procedures. During these treatment processes, emergency medical personnel must stand for a prolonged period. They may also be required to physically support patients, execute bedside aids, carry patients, and turn patients over in bed. These tasks cause the medical personnel to perform straining body movements such as bending and lifting, leading to injuries and discomfort. Additionally, medical personnel may be injured by syringe needles or sharp objects or become infected by contact diseases. When their units prioritize a safety climate and provide them with sufficient protective gear and tools, the chances of emergency medical personnel sustaining harm can be lowered, thus preventing their inability to attend to their positions and accomplish their tasks. This would typically then improve the job performance of the medical personnel. Safety climates affect safety systems and the occupational behaviors associated with personal tasks.^[16]^ Organizations must emphasize a safety climate as a crucial core value. When an enterprise emphasizes having a safe working environment and safety management, the health and safety of its employees are improved, and the employees feel valued; hence, they become more satisfied with their jobs, and their job performance is effectively enhanced.^[17]^ Therefore, in a healthcare system, medical units should prioritize providing a safety climate and enabling their nurses to cohesively maintain occupational safety as a vital goal for the unit operations and medical procedures. Thus, the insecurity and anxiety of nurses can be reduced, thereby improving their job performance.

Contextual performance is defined as the voluntary extra-role job behaviors of an employee that facilitate the growth of the social and psychological environment required to maintain organizational operations with the help of organizational members within the technical cores of the organizations.^[14]^ When the environmental factors were also considered, safety climate (*β* = 0.14, *P* < .001) was significantly associated with the contextual performance of the nurses. Previous study indicated that the correspondence of employees’ expected organizational cultures to their perceived organizational cultures effectively influences their job performance.^[18]^ The job demand and control of a medical department varies with its medical properties and healthcare demands. Job stress, shift systems, and the types of departments significantly affect the job performance of nurses.^[19]^

Based on the results of this study, medical facility managers should establish supportive job environments by using multiple approaches to improve the job performance and physical and psychological health of nurses, thereby promoting the quality of medical care and maximizing the welfare of both nurses and patients. The dimensions may include emphasize the establishment and improvement of safety climates, establish supportive job environments, reduce workload, strengthen social support, and arrange the implementation of physical and psychological health programs.

### 4.2 Methodological consideration

Admittedly, there were several limitations in this study. First, because this study applied the convenience sampling method, the questionnaire survey results may exhibit selection bias. The internal and external validity of the survey results require further examination. Second, the data for this study were acquired from self-descriptions by the interviewees instead of objective measurement. Information bias may have resulted from the data being provided by only the interviewees.

Third, the large data samples may have generated an ecological fallacy when inferred to the individuals. Fourth, this study only obtained subjects from metropolitan 4 teaching hospitals in northern Taiwan as the target population. Therefore, the results of this study should not be extrapolated to hospitals in other regions of Taiwan. Future studies using random sampling of hospitals over a wider range of regions would make the research more discursive. Finally, based on the statistical viewpoint, if there are no missing values and assuming a compound covariance structure, both the GEE and mixed effects models will roughly yield the same results as a traditional GLM repeated measures model. In this study, we assumed missing data completely at random (MCAR) when using GEE model. The mixed-effects repeated measures model may be more appropriated, given its less restrictive assumptions regarding missing values in the further follow-up studies with incomplete information.

## 5 Conclusion

In conclusion, to improve the job performance among nurses in emergency department, it should consider personal psychological and environmental factors.

## Author contributions

Fu-Li Chen, Kuan-Chen Chen, Shy-Yang Chiou, Peter Y. Chen, Man-Li Du, and Tao-Hsin Tung conducted the study and drafted the manuscript. Kuan-Chen Chen and Tao-Hsin Tung participated in the design of the study and performed statistical analyses. Shy-Yang Chiou, Peter Y. Chen, Man-Li Du and Tao-Hsin Tung conceived the study, and participated in its design and coordination. All of the authors read and approved the final manuscript.

**Conceptualization:** Fu-Li Chen.

**Formal analysis:** Kuan-Chen Chen.

**Methodology:** Shy-Yang Chiou.

**Software:** Tao-Hsin Tung.

**Supervision:** Peter Y. Chen, Man-Li Du.

**Writing – original draft:** Fu-Li Chen, Kuan-Chen Chen, Shy-Yang Chiou.

**Writing – review & editing:** Peter Y. Chen, Tao-Hsin Tung.
